# Anlotinib Alone or in Combination With Temozolomide in the Treatment of Recurrent High-Grade Glioma: A Retrospective Analysis

**DOI:** 10.3389/fphar.2021.804942

**Published:** 2021-12-24

**Authors:** Qunying Yang, Chengcheng Guo, Xiaoping Lin, Lili Luo, Zhenqiang He, Fuhua Lin, Ji Zhang, Yinsheng Chen, Xiaobing Jiang, Chao Ke, Yonggao Mou

**Affiliations:** ^1^ Department of Neurosurgery/Neuro-oncology, Sun Yat-sen University Cancer Center, State Key Laboratory of Oncology in South China, Collaborative Innovation Center for Cancer Medicine, Guangzhou, China; ^2^ Department of Nuclear Medicine, Sun Yat-sen University Cancer Center, State Key Laboratory of Oncology in South China, Collaborative Innovation Center for Cancer Medicine, Guangzhou, China

**Keywords:** high-grade glioma, anlotinib, retrospective analysis, efficacy, safety

## Abstract

**Background:** Anlotinib is a multi-target anti-angiogenic agent. This retrospective study aimed to evaluate the efficacy and safety of anlotinib alone or in combination with temozolomide for the treatment of recurrent high-grade glioma.

**Materials and Methods:** The clinical data of patients with recurrent high-grade glioma treated with anlotinib alone or in combination with temozolomide in our cancer center were collected and analyzed. Treatment response was evaluated according to the response assessment for neuro-oncology criteria. Progression-free survival, progression-free survival at 6 months, overall survival, and overall survival at 12 months were evaluated by Kaplan–Meier method and compared by log-rank test.

**Results:** Between August 2019 and December 2020, 31 patients with recurrent high-grade glioma (21 of grade 4 and 10 of grade 3) were enrolled in this study. Seventeen patients received anlotinib alone and 14 received anlotinib plus temozolomide. All patients were heavily treated, the median lines of previous treatments were 2, and the median Karnofsky score was 60. At the data cutoff date, the median progression-free survival was 4.5 months and the progression-free survival at 6 months was 43.5%. The median overall survival was 7.7 months, and the overall survival at 12 months was 26.7%. The progression-free survival at 6 months and the overall survival at 12 months for 21 patients with grade 4 glioma was 40.2 and 27.9%, respectively. The tumor objective response rate was 41.9% in all patients and 33.3% in patients with grade 4 glioma. No grade 3 or worse treatment-related adverse events were recorded during the treatment.

**Conclusion:** Anlotinib alone or in combination with temozolomide showed encouraging efficacy and favorable tolerability in patients with recurrent high-grade glioma who had been heavily treated.

## Introduction

High-grade glioma (HGG) is the most common type of malignant brain tumor in adults, accounting for over 60% of malignant primary brain tumors. HGG is defined as grade 3 gliomas, including anaplastic astrocytoma, anaplastic oligodendroglioma, and anaplastic oligodendrocytoma, and grade 4 gliomas, including pleomorphic glioblastoma, gliosarcoma (GBM), and diffuse midline glioma, by the World Health Organization (WHO) Classification (Ostrom et al., 2015). The estimated 2-year overall survival was 20% after first diagnosis (Marra et al., 2019). To date, the 5-year survival for newly diagnosed GBM is lower than 10% with standard radiotherapy in combination with temozolomide (TMZ) (Stupp et al., 2009). Recurrent HGG is refractory and has limited treatment options. Conventional cytotoxic chemotherapies, such as nitrosourea, irinotecan, cisplatin, carboplatin, etoposide, cyclophosphamide, and ifosfamide, show unsatisfying efficacy and obvious toxicities (Seystahl et al., 2016; Parasramka et al., 2017). Therefore, there is an urgent need for regimens with better efficacy and safety.

HGG is highly vascularized and characterized by the over-expression of vascular endothelial growth factor (VEGF) and other pro-angiogenic cytokines that stimulate the proliferation, migration, and survival of endothelial cells. Bevacizumab was the first anti-angiogenic agent recommended by the NCCN guideline for the treatment of recurrent glioblastoma (Vredenburgh et al., 2007a; Vredenburgh et al., 2007b). Unfortunately, no overall survival benefit was observed using bevacizumab as a single agent or in combination therapy in either first-line or second-line treatment (Lombardi et al., 2017; Wick et al., 2017; Ameratunga et al., 2018). In 2020, regorafenib was recommended for the treatment of recurrent HGG based on a phase 2 trial REGOMA, with a significantly improved overall survival (OS) compared with lomustin (7.4 vs. 5.6 months) (Lombardi et al., 2019). In addition to angiogenesis, the malignant invasiveness of recurrent HGG is associated with other signaling pathways that are involved in tumorigenesis and tumor microenvironment (Lu et al., 2019). Therefore, multi-kinase inhibitors that can regulate various signaling pathways could be used as promising treatment options.

Anlotinib is a novel multi-kinase inhibitor against both tumor angiogenesis and tumor cell proliferation by blocking vascular endothelial growth factor receptor (VEGFR), fibroblast growth factor receptor (FGFR), platelet-derived growth factor receptors (PDGFR), and stem cell factor receptor c-Kit. Anlotinib strongly inhibits the activation of VEGFR2 and VEGFR3, with an IC_50_ of 0.2 and 0.7 nM, 20-fold more potent than sunitinib (Shen et al., 2018; Xie et al., 2018). Molecular modeling indicates that the indole group of anlotinib is located in the hydrophobic region near the Asp-Phe-Gly (DFG) motif, which is a key domain for the regulation of kinase activity, while sunitinib cannot occupy this region. This may partly explain why anlotinib has a higher VEGFR2 binding activity. In addition, the upregulation of FGFR and its ligand FGF can induce tumor angiogenesis and lead to the failure of anti-angiogenic treatment. Anlotinib inhibits the FGFR phosphorylation with an IC_50_ of 11.7 nM, stronger than sunitinib and sorafenib (Lin et al., 2018). In China, anlotinib has been approved for the treatment of non-small cell lung cancer, small cell lung cancer, medullary thyroid cancer, and soft tissue sarcoma (Chi et al., 2018; Han et al., 2018; Cheng et al., 2021; Li et al., 2021).

Anlotinib distributes widely into various body fluids that the mean apparent volume of distribution at steady state in dogs was 12 times as much as the dog volume of total body water. In rats, the exposure levels of anlotinib for lung, liver, kidneys, and heart were 197, 49, 54, and 32 times as much as the systemic exposure level. In tumor-bearing mice, the level of tumor tissue exposure was 13 times as high as the systemic exposure level (Zhong et al., 2018).

The ability of anlotinib to penetrate the blood–brain barrier is not clear. In a *post-hoc* analysis of a phase 3 study on patients with non-small cell lung cancer, anlotinib showed impressive clinical activity in the local control of brain metastases (Jiang et al., 2020). In the 14 patients bearing intracranial lesions treated with anlotinib, the objective response rate and the disease control rate were 14.3 and 85.7%, respectively, and the time to intracranial progression was significantly prolonged compared with patients using placebo. Notably, no cerebral infarction or cerebral hemorrhage events was observed in these patients.

Based on the feasibility of anti-angiogenic strategy in the treatment of HGG and the activity of anlotinib for the treatment of brain metastases, we performed this retrospective study to evaluate the efficacy and safety of anlotinib in patients with recurrent HGG.

## Materials and Methods

### Participants

Patients with pathologically confirmed HGG (WHO 3/4) who had failed the standard first-line and second-line treatment, including surgery, radiation therapy, and adjuvant TMZ treatment, were included in the study. The key eligibility criteria included at least one measurable lesion according to the Response Assessment for Neuro-oncology (RANO) criteria, Karnofsky Performance Status score (KPS) ≥40, age ≤75 years, and adequate organ function revealed by normal blood routine, liver and kidney function, coagulation function, and electrocardiogram. The exclusion criteria included newly diagnosed disease, surgery within 4 weeks or intention for surgical treatment, unhealed wound, history of cerebral hemorrhage or infarction, uncontrollable hypertension, history of myocardial infarction or unstable angina pectoris, stroke, or transient ischemic attack within 6 months. This study was approved by the institutional review board of Sun Yat-sen University Cancer Center (certificate number: B2020-153-01). All patients provided written informed consent.

### Treatment

The patients were administrated with anlotinib orally once daily for 14 days every 3 weeks until disease progression, intolerable toxicities, or death. The initial dose of anlotinib was 12 mg for patients with first recurrent disease and had KPS score ≥60, while it was 10 mg for patients who had repeated recurrence or KPS score <60. The dose can be reduced to 10 or 8 mg if grade 2 hemorrhage or other adverse events of grade 3 or 4 occurred. Combined regimen (TMZ plus anlotinib) was administered to patients who had tumor response and were tolerable to the previous treatment of TMZ with a continuous dose-dense schedule (50 mg/m^2^, QD). Other patients received anlotinib alone.

### Efficacy Evaluation

Magnetic resonance imaging (MRI), contrast-enhanced MRI, and fluid-attenuated inversion recovery (FLAIR) imaging were performed every 3–6 weeks or at any time when disease progression was suspected. Disease assessment was performed according to the RANO criteria (Wen et al., 2010). The primary endpoint was progression-free survival (PFS), defined as the time from the start of anlotinib administration to disease progression or death for any reason. The secondary endpoints included OS (defined as the time from the start of anlotinib treatment until death by any cause), objective response rate (ORR, the proportion of patients who achieved complete or partial response), and disease control rate (DCR, the proportion of patients with complete response, partial response, and stable disease lasting for at least 4 weeks). Adverse events were evaluated according to the National Cancer Institute Common Terminology Criteria for Adverse Events (CTCAE version 5.0).

### Statistical Methods

Statistical analyses were performed with SPSS 26.0. Survival analysis was estimated by Kaplan–Meier method with 95% confidence interval (CI) and compared by log-rank test. Cox regression model was used for univariate and multivariate analyses. Statistical significance was defined as an alpha level of 0.05 (*p* < 0.05).

## Results

### Patient Characteristics

Between August 2019 and December 2020, 31 patients with HGG from the Department of Neurosurgery, Sun Yat-sen University Cancer Center, were included in the study; 19 of them were male and 12 were female. The median age was 42 years (range: 16–75) and the median KPS score was 60 (range: 40–80). A total of 21 patients had grade 4 glioma (19 with GBM and 2 with diffuse midline glioma), and 10 patients had grade 3 glioma (8 with anaplastic astrocytomas and 2 with anaplastic oligodendrogliomas). The lesions of 29 cases were located at the cerebrum and of 2 cases at the cerebellum. Most patients had one or more negative prognostic factors, such as unmethylated MGMT promoter (61.3%) or IDH wild type (71.0%). The proportion of patients with no IDH mutation was similar between those who received anlotinib monotherapy (12/17, 70.6%) or who received a combination regimen (10/14, 71.4%). More patients had unmethylated MGMT promoter in the anlotinib monotherapy subgroup (12/17, 70.6%) compared with that in the combination regimen subgroup (7/14, 50.0%). However, the difference had no statistical significance (*p* = 0.242). Only nine patients had a localized disease, while the other 22 patients had multifocal or disseminated disease. All patients underwent surgery and radiotherapy with concurrent adjuvant TMZ therapy for the primary tumors and had disease recurrence. The previous median medical treatment line was 2 (IQR 2-3, range 1–6). Thirteen patients received bevacizumab, 11 patients received their second surgery, and six patients received at least two radiotherapies before enrollment in this study. The detailed baseline characteristics of the patients are shown in Table 1.

**TABLE 1:** Baseline characteristics of patients.

Characteristics	Number of patients (%)
Gender	
Male	19 (61.3)
Female	12 (38.7)
Age	
≥42 years	17 (54.8)
<42 years	14 (45.2)
KPS score	
≥60	20 (64.5)
<60	11 (35.5)
Grade of histology	
4	21 (67.7)
3	10 (32.3)
Tumor location	
Multifocal/dissemination	22 (71.0)
Focal	9 (29.0)
MGMT promoter status	
Methylation	12 (38.7)
Unmethylation	19 (61.3)
IDH status	
Wild type	22 (71.0)
Mutation	9 (29.0)
1p/19q deletion	
Positive	7 (22.6)
Negative	16 (51.6)
Unknown	8 (25.8)
Line of previous treatment for recurrent disease	
0	7 (22.6)
1	13 (41.9)
≥2	11 (35.5)
Previous anti-angiogenic agents	
Yes	13 (41.9)
No	18 (58.1)
Previous re-operation	
Yes	11 (35.5)
No	20 (64.5)
Previous re-radiation	
Yes	6 (19.4)
No	25 (80.6)
Study treatment	
Anlotinib plus temozolomide	14 (45.2)
Anlotinib	17 (54.8)

KPS, Karnofsky Performance Status score; MGMT, O6-methylguanine-DNA methyltransferase; IDH, isocitrate dehydrogenase.

### Treatment

Seventeen patients received anlotinib alone and 14 received a combination regimen with TMZ. The initial dose of anlotinib was 12 mg QD for 10 patients and 10 mg QD for 21 patients. One patient who had a combined regimen experienced a dose reduction (from 10 to 8 mg) due to hypertension, headache, nausea, and fatigue. At the data cutoff date on May 31, 2021, the median duration of follow-up was 15.9 months (95% CI: 10.3–21.5). A total of 26 patients developed disease progression (9 with grade 3 HGG and 17 with grade 4 HGG). Then, 21 patients were dead (7 with grade 3 HGG and 14 with grade 4 HGG). The total treatment cycles for the 31 patients were 184, and the median duration of treatment was 4 cycles (IQR 2.0–6.5; range 1–21).

### Efficacy

**FIGURE 1:**
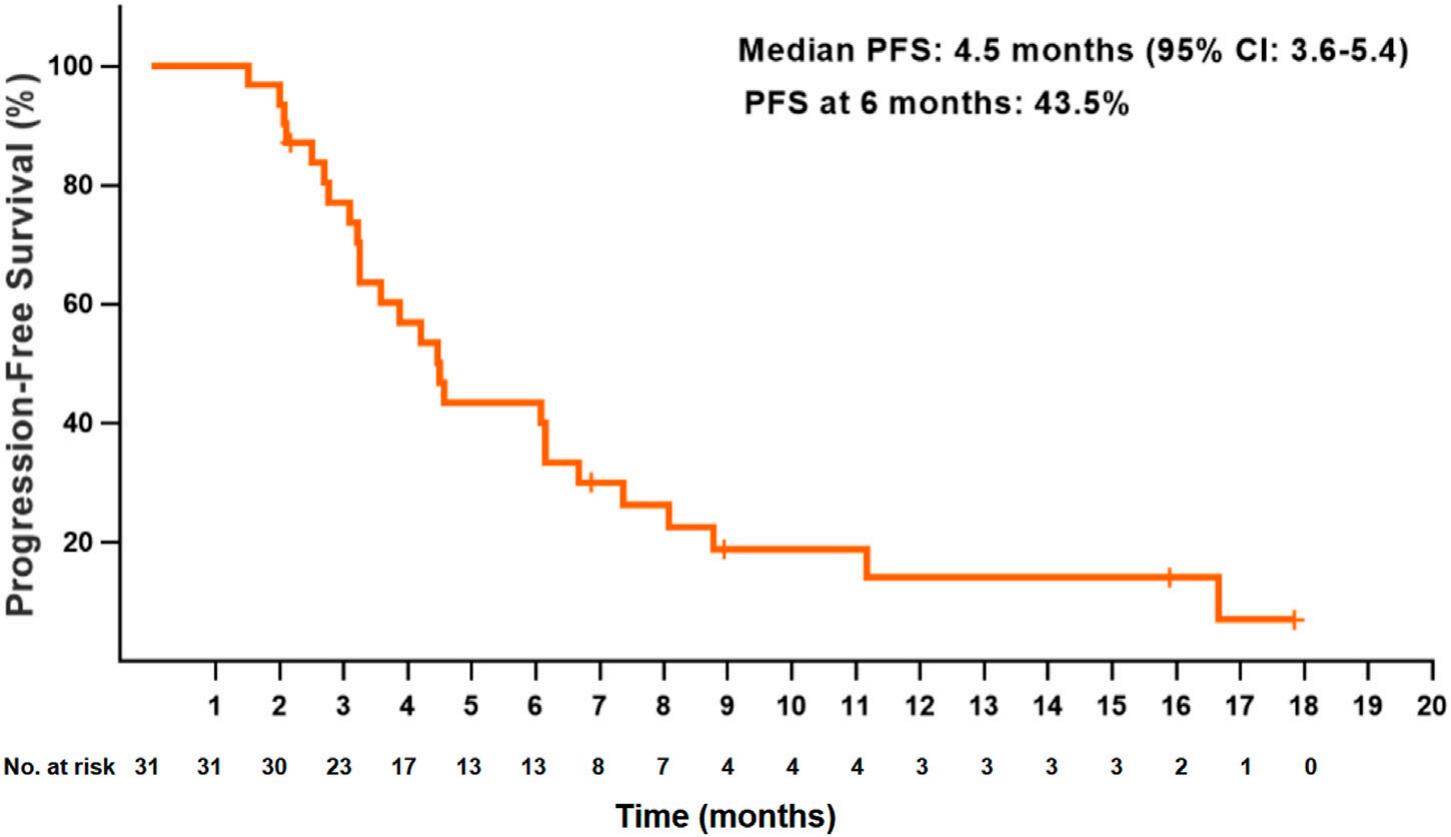
Kaplan–Meier plot of progression-free survival in all patients.

**FIGURE 2:**
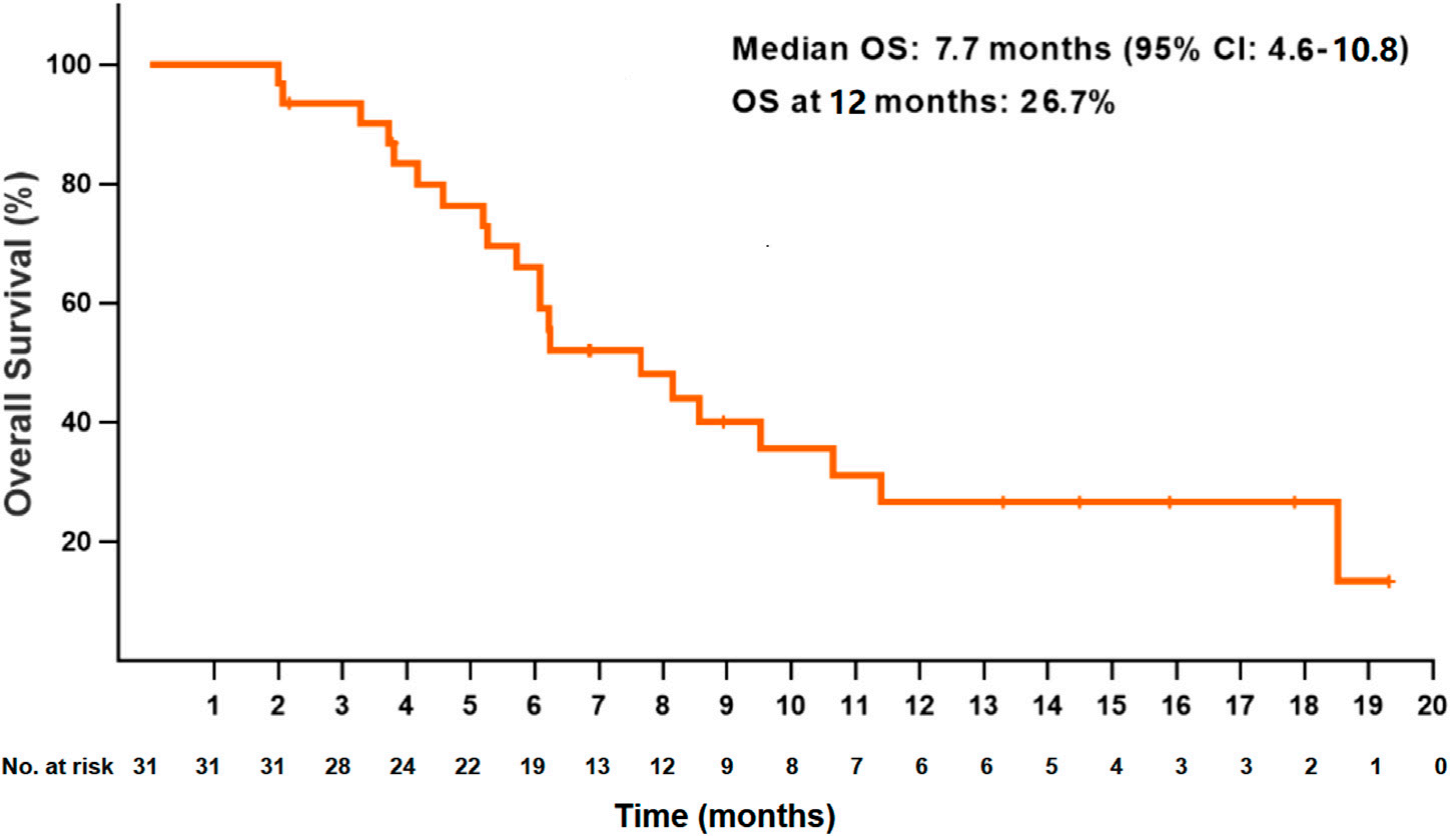
Kaplan–Meier plot of overall survival in all patients.

At the data cutoff date, the median PFS for all patients was 4.5 months (95% CI: 3.6–5.4) (Figure 1). The PFS at 6 and 12 months were 43.5 and 14.1%, respectively. The median OS was 7.7 months (95% CI: 4.6–10.8), and the OS at 12 months was 26.7% (Figure 2). Thirteen patients (41.9%) achieved a partial response, and 11 patients (35.5%) had a stable disease. The ORR and DCR were 41.9 and 77.4%, respectively, for all patients. Rapid and long duration of response were observed. The median time to tumor response and the median duration of response were 1.7 months (95% CI: 0.58–2.8) and 6.1 months, respectively (95% CI: 2.2–10.0).

**FIGURE 3:**
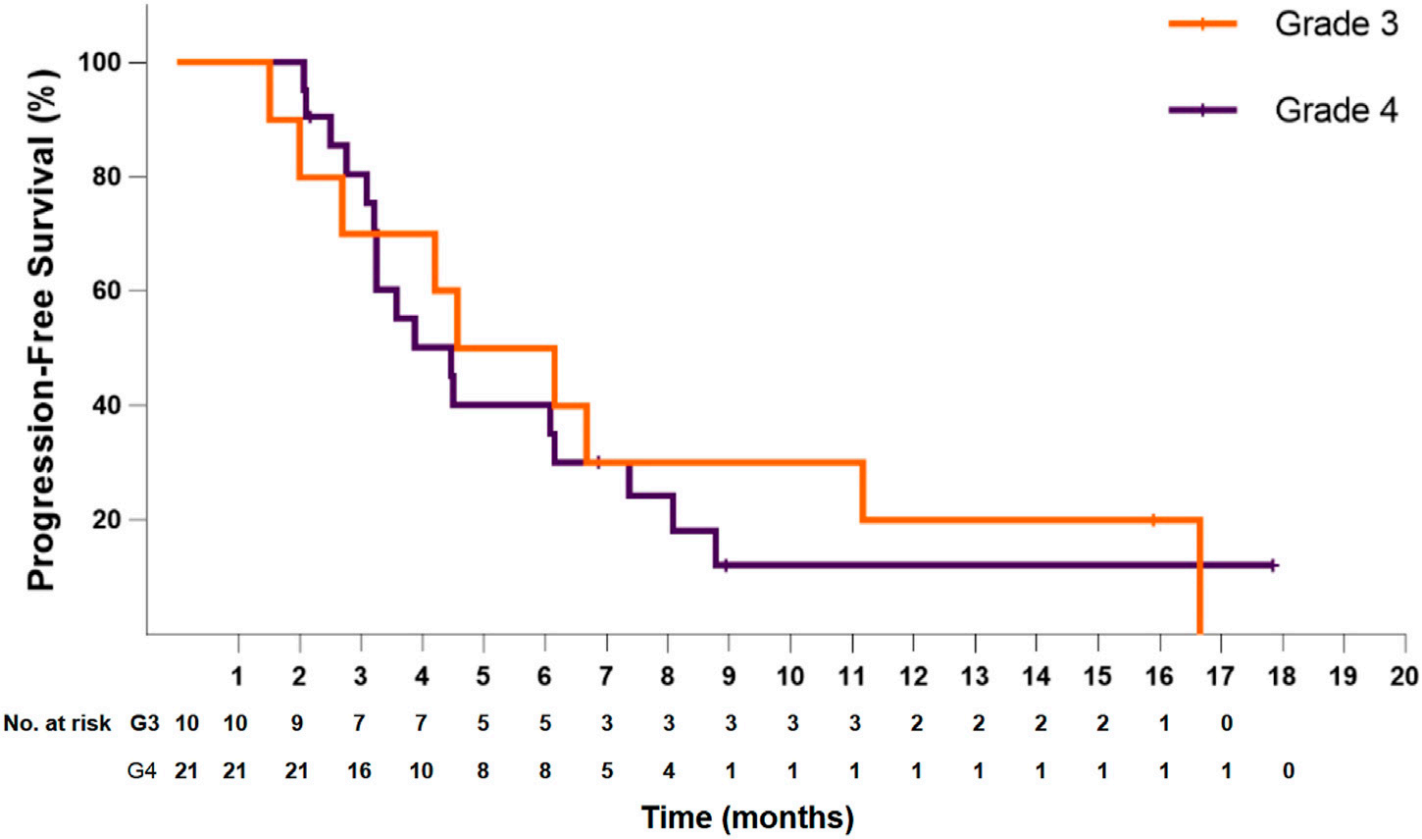
Kaplan–Meier plot of progression-free survival for patients with grade 3 (orange) or grade 4 (violet) disease.

**FIGURE 4:**
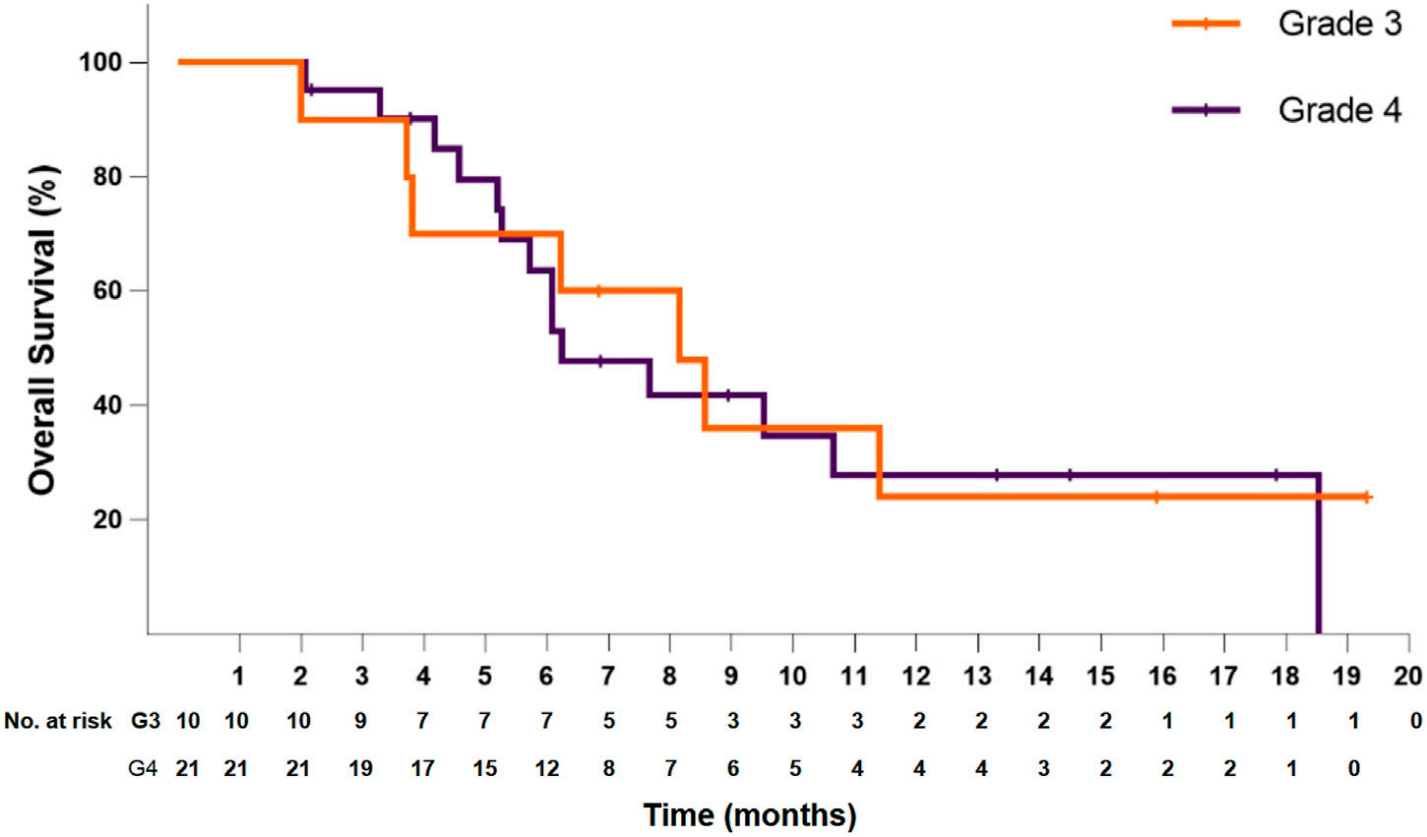
Kaplan–Meier plot of overall survival for patients with grade 3 (orange) or grade 4 (violet) disease.

In the 21 patients with grade 4 disease, the median PFS was 4.5 months (95% CI: 3.1–5.8), and the PFS at 6 months was 40.2%. The median OS was 6.2 months (95% CI: 4.3–8.2), and the OS at 12 months was 27.9%. In the 10 patients with grade 3 disease, the median PFS was 4.6 months (95% CI: 1.6–7.6), and the PFS at 6 months was 50.0%. The median OS was 8.1 months (95% CI: 5.0–11.3), and the OS at 12 months was 24.0% (Figures 3, 4). The ORR and DCR for patients with grade 4 disease were 33.3 and 71.4%, respectively. For the 10 patients with grade 3 disease, six patients showed a tumor response, and the ORR was up to 60%. The DCR was 90%. The combination treatment showed a higher tumor response rate and more survival benefits than anlotinib alone. The ORR was 57.1 and 29.4%, respectively, for the patients in the two subgroups. The median PFS was 6.1 months (95% CI: 3.0–9.2) versus 4.2 months [95% CI: 2.4–6.0; *p* = 0.29, HR = 0.66 (95% CI: 0.30–1.44)]. The median OS was 10.6 months (95% CI: 6.8–14.5) and 6.1 months (95% CI: 5.4–6.8), respectively, in the two groups, with a hazard ratio of 0.63 (95% CI: 0.26–1.50).

The results for the sub-analysis are shown in Table 2. In a univariate analysis, the patients with KPS score ≥60 had a significant PFS benefit from treatment than those with KPS score <60 (6.7 *versus* 3.3 months; *p* < 0.001). Patients with multifocal or disseminated disease had a significantly higher progression risk than those with a localized disease, and the median PFS was 3.2 *versus* 8.1 months (*p* = 0.009). The previous bevacizumab treatment significantly diminished the efficacy of treatment [HR = 2.2 (95% CI: 1.00–4.83), *p* = 0.044]. However, these results must be interpreted cautiously since 12 (66.7%) of 18 patients who were previously bevacizumab-naive had received a combined regimen with TMZ, while only 2 (15.4%) of 13 patients who were previously on bevacizumab received a combined regimen. A trend of longer PFS was detected in patients with MGMT promoter methylation [HR = 0.68 (95% CI: 0.31–1.53)] or IDH mutation [HR = 0.74 (95% CI: 0.31–1.74)].

**TABLE 2:** Results of the univariate analysis.

Characteristics	Number of patients	Median PFS	*p*-value	HR (95% CI)
		Months (95% CI)		
Gender				
Male	19	6.1 (3.1, 9.2)	0.76	1.14 (0.50, 2.62)
Female	12	4.2 (2.7, 5.7)	—	—
Age				
≥42 years	17	4.6 (2.7, 6.5)	0.93	1.04 (0.47, 2.28)
<42 years	14	4.2 (3.1, 5.3)	—	—
KPS score				
≥60	20	6.7 (3.7, 9.6)	<0.001	0.18 (0.068, 0.481)
<60	11	3.3 (2.5, 4.0)	—	—
Histology				
Grade 4	21	4.5 (3.1, 5.8)	0.70	1.18 (0.52, 2.68)
Grade 3	10	4.6 (1.6, 7.6)	—	—
Tumor location				
Multifocal/dissemination	22	3.2 (2.7, 3.8)	0.009	3.23 (1.27, 8.21)
Focal	9	8.1 (6.3, 9.9)	—	—
MGMT promoter status				
Methylation	12	6.1 (2.0, 10.3)	0.35	0.68 (0.31, 1.53)
unmethylation	19	4.5 (2.6, 6.3)	—	—
IDH status				
Mutation	9	6.1 (0.48, 11.8)	0.49	0.74 (0.31, 1.74)
Wild type	22	4.5 (3.1, 5.8)	—	—
Previous anti-angiogenesis				
Yes	13	3.3 (2.4, 4.1)	0.044	2.2 (1.00, 4.83)
No	18	6.1 (2.8, 9.5)	—	—
Study treatment				
Anlotinib plus TMZ	14	6.1 (3.0, 9.2)	0.29	0.66 (0.30, 1.44)
Anlotinib	17	4.2 (2.4. 6.0)	—	—

KPS, Karnofsky Performance Status score; MGMT, O^6^-methylguanine-DNA methyltransferase; IDH, isocitrate dehydrogenase; TMZ, temozolomide.

The multivariate analysis for PFS involved tumor grades, KPS score, lesion diffusion (localized or disseminated disease), and treatment regimen. Only the KPS score was found as an independent prognostic factor, in which patients with a higher KPS score (≥60) had a significantly longer PFS compared with their counterparts [HR = 0.22 (95% CI: 0.079–0.634), *p* = 0.005]. Multifocal or disseminated disease showed an obviously negative impact on the prognosis, and the HR for PFS was 2.44 (95% CI: 0.85–7.04, *p* = 0.099) compared with localized disease. Treatment with the combined regimen achieved more PFS benefit than monotherapy. However, the difference was not significant. Similarly, the multivariate analysis for OS involved four factors as mentioned above. The KPS score and lesion diffusion were two key prognostic factors, and the HR was 0.29 (95% Cl: 0.11–0.77; *p* = 0.013; ≥60 *vs*. < 60) and 3.73 (95% CI: 0.94–14.82; *p* = 0.061; disseminated *vs*. localized).

### Safety

All treatment-related adverse events were grade 1 or 2 (Table 3). The most common treatment-related adverse events for patients treated with anlotinib alone included hypertension (41.2%), hypertriglyceridemia (17.6%), and oral mucositis (17.6%). The most common treatment-related adverse events caused by the combination treatment included gastrointestinal reactions (64.3%), leukopenia (64.3%), hypertension (57.1%), anemia (57.1%), and liver function impairment (50%). One patient had a dose reduction of anlotinib from 10 to 8 mg because of hypertension, nausea, and fatigue. No treatment-related discontinuation of anlotinib occurred.

**TABLE 3:** The most common treatment-related adverse events.

	Grades 1 and 2		
	All (*n* = 31)	Anlotinib and TMZ (*n* = 14)	Anlotinib (*n* = 17)
Hypertension, *n* (%)	15 (48.4)	8 (57.1)	7 (41.2)
Gastrointestinal reactions, *n* (%)	11 (35.5)	9 (64.3)	2 (11.8)
Leukopenia, *n* (%)	11 (35.5)	9 (64.3)	2 (11.8)
Anemia, *n* (%)	8 (25.8)	8 (57.1)	0
Oral mucositis, *n* (%)	7 (22.6)	4 (28.6)	3 (17.6)
Liver function impairment, *n* (%)	7 (22.6)	7 (50)	0
Hypertriglyceridemia, *n* (%)	5 (16.1)	2 (14.3)	3 (17.6)
Proteinuria, *n* (%)	4 (12.9)	3 (21.4)	1 (5.9)
Hand–foot reaction, *n* (%)	4 (12.9)	3 (21.4)	1 (5.9)
Electrocardiographic abnormality, *n* (%)	4 (12.9)	4 (28.6)	0
Thrombocytopenia, *n* (%)	3 (9.7)	3 (21.4)	0
Bleeding, *n* (%)	2 (6.5)	1 (7.1)	1 (5.9)
Hypothyroidism, *n* (%)	2 (6.5)	1 (7.1)	1 (5.9)

## Discussion

The prognosis for patients with HGG was poor, especially for those who had been heavily treated. The median PFS for patients with recurrent malignant glioma who received dose-intense TMZ was only 3.5 months (Perry et al., 2010). Other than chemotherapy, the treatment approach for recurrent HGG is limited. Anti-angiogenesis is the only recognized targeted therapy for HGG (NCCN, 2021). Anlotinib is a multi-kinase inhibitor targeting both angiogenesis and tumor cell proliferation by blocking VEGFR, PDGFR, FGFR, and c-Kit. To our knowledge, this is the first clinical study for anlotinib in patients with recurrent HGG. In this retrospective study, anlotinib, as a single agent or in combination with TMZ, showed a median PFS of 4.5 months and a median OS of 7.7 months in patients with recurrent HGG. Twenty-one patients enrolled in our study have grade 4 glioma (19 patients with GMB and 2 patients with diffuse midline glioma). The median PFS and OS for these patients were 4.5 and 6.2 months, respectively. The PFS at 6 months and the OS at 12 months were 40.2 and 27.9%, respectively. These results exceeded the data (35.7 and 28.6%) reported in the study of TMZ re-challenge even if ignoring the bias that the patients in the TMZ re-challenge study had better baseline characteristics, such as no previous treatment for recurrent disease, better physical condition (Eastern Cooperative Oncology Group performance status ≤1), and higher proportion of MGMT promoter methylation (42%) (Perry et al., 2010).

With the approval of bevacizumab, treatment for HGG has entered the era of targeted therapy. In the BRAIN study for recurrent GBM, the ORR for bevacizumab alone and bevacizumab plus irinotecan was 28.2 and 37.8%, respectively, and the PFS was 4.2 and 5.6 months, respectively (Friedman et al., 2009). In the two large-scale phase 3 studies evaluating the efficacy of bevacizumab and lomustine in GBM as the second line of treatment, the median PFS was 4 and 4.2 months, respectively (Taal et al., 2014; Wick et al., 2017). The results of our study were comparable with the above-mentioned studies on bevacizumab. The ORR for patients with grade 4 glioma was 33.3%, and the median PFS for patients with grade 4 glioma was 4.5 months. However, the subjects enrolled in our study had poor baseline characteristics. The median lines of previous treatments were 2 (IQR: 2, 6), while the eligible patients in other studies usually just experienced only one or two lines of treatment. The median KPS score was 60, worse than that of most of the other previous studies. In addition, most patients (22, 71.0%) in our study had disseminated disease. A similar study had been performed by Verhoeff *et al*. to evaluate the efficacy of bevacizumab and dose-intense TMZ (50 mg/m^2^ once daily) in recurrent HGG. This study included eight patients with grade 3 glioma and 15 with grade 4 glioma, similar to our study. The ORR was only 20%. The PFS at 6 months was as low as 17.4%, and the median OS was only 17.1 weeks in the study (Verhoeff et al., 2010).

Regorafenib is a multi-kinase inhibitor against VEGFR1-3, angiopoietin-1 receptor (TIE2), KIT, RET, PDGFR, FGFR, and others (Wilhelm et al., 2011). In the REGOMA study which compared regorafenib monotherapy with lomustine for patients with GBM after the first recurrence, regorafenib achieved a significant OS benefit with the median OS of 7.4 *versus* 5.6 months. The median PFS was 2 *versus* 1.9 months (Lombardi et al., 2019). This study indicated the advantages of multi-target tyrosine kinase inhibitors. However, some queries arose because the patients who received lomustine showed abnormally poor overall survival and progression-free survival.

Anlotinib shares similar targets with regorafenib but has a higher potency of inhibition. The IC_50_ for VEGFR2 inhibition was 0.2 *versus* 4.2 nM. Anlotinib inhibits the activation of FGFR by blocking the phosphorylation of FGFR1 with an inhibitory rate of 45.0% (p-FGFR1/ FGFR1) at 1 μM and shows an IC_50_ value of 25 nM in AN3Ca cells overexpressing a FGFR2 mutant protein in another assay (Lin et al., 2018). Many studies have proved that the FGFR signaling pathway played an important role in the progression of GMB and that the FGFR1 overexpression was associated with the higher grade of disease (Jimenez-Pascual et al., 2020). So, the blockade of FGFR signaling may be beneficial, and the FGFR inhibitors had been investigated (Bahleda et al., 2019). Regorafenib can also target FGFR. However, the activity is moderate, and the IC_50_ is 202 nM (Wilhelm et al., 2011). In fact, the clinical outcomes were inconsistent with the higher activity of anlotinib *in vitro*.

Distinctly from other studies, our study enrolled 13 patients who had previously received treatment with anti-angiogenic agents, which obviously affected the PFS benefit of patients (median PFS: 3.3 *vs*. 6.1 months). The baseline physical conditions of patients who previously received anti-angiogenic agents were poorer compared with that of all patients. The median KPS score was 50, and the median number of previous lines of treatments was 3. Most of these heavily treated patients (11 of 13) received anlotinib alone, which may be due to the ineffectiveness of or intolerance to TMZ. Overall, the 3.3-month PFS indicated the feasibility of anlotinib as a single agent for salvage treatment. On the other hand, earlier treatment with anlotinib in combination with chemotherapy may bring greater survival benefit to patients.

In patients treated with the monotherapy subgroup, more patients had unmethylated MGMT promoter (12/17, 70.6%) compared with those in the combination regimen (7/14, 50.0%). This may lead to a slightly unfavorable bias to patients with monotherapy when comparing the treatment efficacy since the unmethylated MGMT promoter is associated with poor prognosis. The possible reason for the higher proportions of patients with unmethylated MGMT promoter in the monotherapy subgroup was that the patients with methylated MGMT promoter were usually sensitive to TMZ in the previous treatment and were arranged to receive a combination regimen according to our protocol.

Treatment with anlotinib showed favorable tolerability in this study. Dose reduction only occurred in one patient (from 12 to 8 mg), and the symptoms were relieved after dose adjustment. To our surprise, no grade 3 or 4 treatment-related AE was observed. In addition to the favorable safety and tolerance profile of anlotinib, this result can also be attributed to several other reasons. Firstly, a flexible initial dose of anlotinib, adjusted according to the physical condition of the patients, was used. It has been reported that the efficacy of bevacizumab at a dose of 5 mg/kg every 3 weeks was comparable with 10 mg/kg every 2 weeks while the toxicity was reduced (Bahleda et al., 2019). Secondly, the duration of treatment was relatively short. The toxicity of the treatment regimen was related to the treatment course. In the study of anlotinib as the second-line treatment for renal clear cell carcinoma (median PFS: 8.5 months) and as first-line treatment for medullary thyroid cancer (median PFS: 20.7 months), the incidence of dose adjustment was 11.9 and 32.2%, respectively (Ma et al., 2020; Li et al., 2021). Thirdly, this is a retrospective study, the adverse events might be underestimated for the incomplete records. The most common TRAE caused by anlotinib was hypertension (41.2%). Most hematological adverse events occurred in patients with the treatment combination of anlotinib and TMZ.

Our studies have some limitations. Firstly, this is a retrospective single-arm study with a small sample size. Secondly, patients with glioma of both grade 3 and 4 were enrolled and treated with a different regimen, which complicated the interpretation of the results. Nevertheless, the aim of this study was to preliminarily evaluate the efficacy and safety of anlotinib alone or in combination with TMZ in the treatment of recurrent HGG. Promising outcomes have been obtained. Based on these results, a prospective phase II study to evaluate the efficacy and safety of the combination regimen with anlotinib and TMZ in patients with GBM after first recurrence is ongoing.

## Conclusion

This study showed the promising efficacy and favorable tolerability of anlotinib alone and in combination with TMZ in the treatment of patients with recurrent HGG. Encouraging survival benefit and tumor response were observed. These results indicated that anlotinib with or without TMZ is a promising treatment option for patients with recurrent HGG.

## Data Availability

The original contributions presented in the study are included in the article/supplementary material. Further inquiries can be directed to the corresponding authors.

## References

[B1] Ameratunga M. Pavlakis N. Wheeler H. Grant R. Simes J. Khasraw M. (2018). Anti-angiogenic Therapy for High-Grade Glioma. Cochrane Database Syst. Rev. 11, CD008218. 10.1002/14651858.CD008218.pub4 30480778 PMC6516839

[B2] Bahleda R. Italiano A. Hierro C. Mita A. Cervantes A. Chan N. (2019). Multicenter Phase I Study of Erdafitinib (JNJ-42756493), Oral Pan-Fibroblast Growth Factor Receptor Inhibitor, in Patients with Advanced or Refractory Solid Tumors. Clin. Cancer Res. 25, 4888–4897. 10.1158/1078-0432.CCR-18-3334 31088831

[B3] Cheng Y. Wang Q. Li K. Shi J. Liu Y. Wu L. (2021). Anlotinib vs Placebo as Third- or Further-Line Treatment for Patients with Small Cell Lung Cancer: a Randomised, Double-Blind, Placebo-Controlled Phase 2 Study. Br. J. Cancer 125 (3), 366–371. 10.1038/s41416-021-01356-3 34006926 PMC8329046

[B4] Chi Y. Yao Y. Wang S. Huang G. Cai Q. Shang G. (2018). Anlotinib for Metastasis Soft Tissue Sarcoma: A Randomized, Double-Blind, Placebo-Controlled and Multi-Centered Clinical Trial. Jco 36 (15_Suppl. l), 11503. 10.1200/jco.2018.36.15_suppl.11503

[B5] Friedman H. S. Prados M. D. Wen P. Y. Mikkelsen T. Schiff D. Abrey L. E. (2009). Bevacizumab Alone and in Combination with Irinotecan in Recurrent Glioblastoma. J. Clin. Oncol. 27, 4733–4740. 10.1200/JCO.2008.19.8721 19720927

[B6] Han B. Li K. Wang Q. Zhang L. Shi J. Wang Z. (2018). Effect of Anlotinib as a Third-Line or Further Treatment on Overall Survival of Patients with Advanced Non-small Cell Lung Cancer: The ALTER 0303 Phase 3 Randomized Clinical Trial. JAMA Oncol. 4, 1569–1575. 10.1001/jamaoncol.2018.3039 30098152 PMC6248083

[B7] Jiang S. Liang H. Liu Z. Zhao S. Liu J. Xie Z. (2020). The Impact of Anlotinib on Brain Metastases of Non-small Cell Lung Cancer: Post Hoc Analysis of a Phase III Randomized Control Trial (ALTER0303). Oncologist 25, e870–e874. 10.1634/theoncologist.2019-0838 32077550 PMC7216446

[B8] Jimenez-Pascual A. Mitchell K. Siebzehnrubl F. A. Lathia J. D. (2020). FGF2: a Novel Druggable Target for Glioblastoma? Expert Opin. Ther. Targets 24, 311–318. 10.1080/14728222.2020.1736558 32174197 PMC9075824

[B9] Li D. Chi Y. Chen X. Ge M. Zhang Y. Guo Z. (2021). Anlotinib in Locally Advanced or Metastatic Medullary Thyroid Carcinoma: A Randomized, Double-Blind Phase IIB Trial. Clin. Cancer Res. 27 (13), 3567–3575. 10.1158/1078-0432.CCR-20-2950 33832949

[B10] Lin B. Song X. Yang D. Bai D. Yao Y. Lu N. (2018). Anlotinib Inhibits Angiogenesis via Suppressing the Activation of VEGFR2, PDGFRβ and FGFR1. Gene 654, 77–86. 10.1016/j.gene.2018.02.026 29454091

[B11] Lombardi G. De Salvo G. L. Brandes A. A. Eoli M. Rudà R. Faedi M. (2019). Regorafenib Compared with Lomustine in Patients with Relapsed Glioblastoma (REGOMA): a Multicentre, Open-Label, Randomised, Controlled, Phase 2 Trial. Lancet Oncol. 20, 110–119. 10.1016/S1470-2045(18)30675-2 30522967

[B12] Lombardi G. Pambuku A. Bellu L. Farina M. Della Puppa A. Denaro L. (2017). Effectiveness of Antiangiogenic Drugs in Glioblastoma Patients: A Systematic Review and Meta-Analysis of Randomized Clinical Trials. Crit. Rev. Oncol. Hematol. 111, 94–102. 10.1016/j.critrevonc.2017.01.018 28259301

[B13] Lu Q. R. Qian L. Zhou X. (2019). Developmental Origins and Oncogenic Pathways in Malignant Brain Tumors. Wiley Interdiscip. Rev. Dev. Biol. 8, e342. 10.1002/wdev.342 30945456 PMC6565468

[B14] Ma J. Song Y. Shou J. Bai Y. Li H. Xie X. (2020). Anlotinib for Patients with Metastatic Renal Cell Carcinoma Previously Treated with One Vascular Endothelial Growth Factor Receptor-Tyrosine Kinase Inhibitor: A Phase 2 Trial. Front. Oncol. 10, 664. 10.3389/fonc.2020.00664 32457838 PMC7221023

[B15] Marra J. S. Mendes G. P. Yoshinari G. H. Jr da Silva Guimarães F. Mazin S. C. de Oliveira H. F. (2019). Survival after Radiation Therapy for High-Grade Glioma. Rep. Pract. Oncol. Radiother. 24, 35–40. 10.1016/j.rpor.2018.09.003 30337846 PMC6187089

[B16] NCCN (2021). NCCN Guidelines Central Nervous System Cancers 2021 V1. Pennsylvania: NCCN.

[B17] Ostrom Q. T. Gittleman H. Fulop J. Liu M. Blanda R. Kromer C. (2015). CBTRUS Statistical Report: Primary Brain and Central Nervous System Tumors Diagnosed in the United States in 2008-2012. Neuro Oncol. 17 (Suppl. 4), iv1–iv62. 10.1093/neuonc/nov189 26511214 PMC4623240

[B18] Parasramka S. Talari G. Rosenfeld M. Guo J. Villano J. L. (2017). Procarbazine, Lomustine and Vincristine for Recurrent High-Grade Glioma. Cochrane Database Syst. Rev. 7, CD011773. 10.1002/14651858.CD011773.pub2 28744879 PMC6483418

[B19] Perry J. R. Bélanger K. Mason W. P. Fulton D. Kavan P. Easaw J. (2010). Phase II Trial of Continuous Dose-Intense Temozolomide in Recurrent Malignant Glioma: RESCUE Study. J. Clin. Oncol. 28, 2051–2057. 10.1200/JCO.2009.26.5520 20308655

[B20] Seystahl K. Wick W. Weller M. (2016). Therapeutic Options in Recurrent Glioblastoma--An Update. Crit. Rev. Oncol. Hematol. 99, 389–408. 10.1016/j.critrevonc.2016.01.018 26830009

[B21] Shen G. Zheng F. Ren D. Du F. Dong Q. Wang Z. (2018). Anlotinib: a Novel Multi-Targeting Tyrosine Kinase Inhibitor in Clinical Development. J. Hematol. Oncol. 11, 120. 10.1186/s13045-018-0664-7 30231931 PMC6146601

[B22] Stupp R. Hegi M. E. Mason W. P. van den Bent M. J. Taphoorn M. J. Janzer R. C. (2009). European Organisation for Research and Treatment of Cancer Brain Tumour and Radiation Oncology Groups; National Cancer Institute of Canada Clinical Trials GroupEffects of Radiotherapy with Concomitant and Adjuvant Temozolomide versus Radiotherapy Alone on Survival in Glioblastoma in a Randomised Phase III Study: 5-year Analysis of the EORTC-NCIC Trial. Lancet Oncol. 10, 459–466. 10.1016/S1470-2045(09)70025-7 19269895

[B23] Taal W. Oosterkamp H. M. Walenkamp A. M. Dubbink H. J. Beerepoot L. V. Hanse M. C. (2014). Single-agent Bevacizumab or Lomustine versus a Combination of Bevacizumab Plus Lomustine in Patients with Recurrent Glioblastoma (BELOB Trial): a Randomised Controlled Phase 2 Trial. Lancet Oncol. 15, 943–953. 10.1016/S1470-2045(14)70314-6 25035291

[B24] Verhoeff J. J. C. Lavini C. van Linde M. E. Stalpers L. J. A. Majoie C. B. L. M. Reijneveld J. C. (2010). Bevacizumab and Dose-Intense Temozolomide in Recurrent High-Grade Glioma. Ann. Oncol. 21, 1723–1727. 10.1093/annonc/mdp591 20064829

[B25] Vredenburgh J. J. Desjardins A. Herndon J. E. 2nd Dowell J. M. Reardon D. A. Quinn J. A. (2007). Phase II Trial of Bevacizumab and Irinotecan in Recurrent Malignant Glioma. Clin. Cancer Res. 13, 1253–1259. 10.1158/1078-0432.CCR-06-2309 17317837

[B26] Vredenburgh J. J. Desjardins A. Herndon J. E. 2nd Marcello J. Reardon D. A. Quinn J. A. (2007). Bevacizumab Plus Irinotecan in Recurrent Glioblastoma Multiforme. J. Clin. Oncol. 25, 4722–4729. 10.1200/JCO.2007.12.2440 17947719

[B27] Wen P. Y. Macdonald D. R. Reardon D. A. Cloughesy T. F. Sorensen A. G. Galanis E. (2010). Updated Response Assessment Criteria for High-Grade Gliomas: Response Assessment in Neuro-Oncology Working Group. J. Clin. Oncol. 28 (11), 1963–1972. 10.1200/JCO.2009.26.3541 20231676

[B28] Wick W. Gorlia T. Bendszus M. Taphoorn M. Sahm F. Harting I. (2017). Lomustine and Bevacizumab in Progressive Glioblastoma. N. Engl. J. Med. 377, 1954–1963. 10.1056/NEJMoa1707358 29141164

[B29] Wilhelm S. M. Dumas J. Adnane L. Lynch M. Carter C. A. Schütz G. (2011). Regorafenib (BAY 73-4506): a New Oral Multikinase Inhibitor of Angiogenic, Stromal and Oncogenic Receptor Tyrosine Kinases with Potent Preclinical Antitumor Activity. Int. J. Cancer 129, 245–255. 10.1002/ijc.25864 21170960

[B30] Xie C. Wan X. Quan H. Zheng M. Fu L. Li Y. (2018). Preclinical Characterization of Anlotinib, a Highly Potent and Selective Vascular Endothelial Growth Factor Receptor-2 Inhibitor. Cancer Sci. 109, 1207–1219. 10.1111/cas.13536 29446853 PMC5891194

[B31] Zhong C. C. Chen F. Yang J. L. Jia W. W. Li L. Cheng C. (2018). Pharmacokinetics and Disposition of Anlotinib, an Oral Tyrosine Kinase Inhibitor, in Experimental Animal Species. Acta Pharmacol. Sin 39 (6), 1048–1063. 10.1038/aps.2017.199 29620050 PMC6256268

